# Intraoperative responses of motor evoked potentials to the novel intravenous anesthetic remimazolam during spine surgery: a report of two cases

**DOI:** 10.1186/s40981-020-00401-z

**Published:** 2020-12-09

**Authors:** Takashi Kondo, Yukari Toyota, Soshi Narasaki, Tomoyuki Watanabe, Hirotsugu Miyoshi, Noboru Saeki, Yasuo M. Tsutsumi

**Affiliations:** 1 Department of Anesthesiology and Critical Care, Graduate School of Biomedical and Health Sciences, Hiroshima University, 1-2-3 Kasumi, Minami-ku, Hiroshima, 734-8551 Japan; 2 Department of Anesthesiology and Critical Care, Hiroshima University Hospital, 1-2-3 Kasumi, Minami-ku, Hiroshima, 734-8551 Japan

**Keywords:** Remimazolam, Motor evoked potentials, Spine surgery

## Abstract

**Background:**

Remimazolam is a novel short-acting benzodiazepine characterized by metabolism independent from organ function. We report intraoperative MEP responses of two patients who underwent spine surgery under general anesthesia using remimazolam.

**Case presentation:**

In case 1, MEP monitoring was successfully performed with the use of a fixed dose of remimazolam at 0.5 mg/kg/h and remifentanil at 0.2 μg/kg/min. In case 2, an increasing dose of remimazolam from 0.5 to 1.5 mg/kg/h during the operation did not affect MEP signals. In both cases, remimazolam was titrated to maintain the values of entropy electroencephalogram (EEG) monitoring at 40–60.

**Conclusions:**

General anesthesia using remimazolam and remifentanil can be a valuable alternative for spine surgery with MEP monitoring by EEG to assess the optimal dose.

## Background

Spinal cord injury is an important complication of major spine surgery. To prevent intraoperative spinal cord damage, neurophysiologic monitoring of motor evoked potentials (MEP) is recommended during surgical procedures [1]. Most anesthetic agents other than opioids depress MEP responses [2]; therefore, adequate use of anesthetics is required to evaluate intraoperative changes in MEP signals with minimal interference. Remimazolam, a novel intravenous anesthetic, is a short-acting benzodiazepine characterized by metabolism independent of organ function [3]. The conventionally used benzodiazepine midazolam can be safely used during surgery with MEP monitoring [4]. However, the efficacy of remimazolam during surgery with MEP monitoring remains unclear. Here, we report intraoperative MEP responses of two patients who underwent spine surgery under general anesthesia using remimazolam.

## Case presentations

### Case 1

**Fig. 1:**
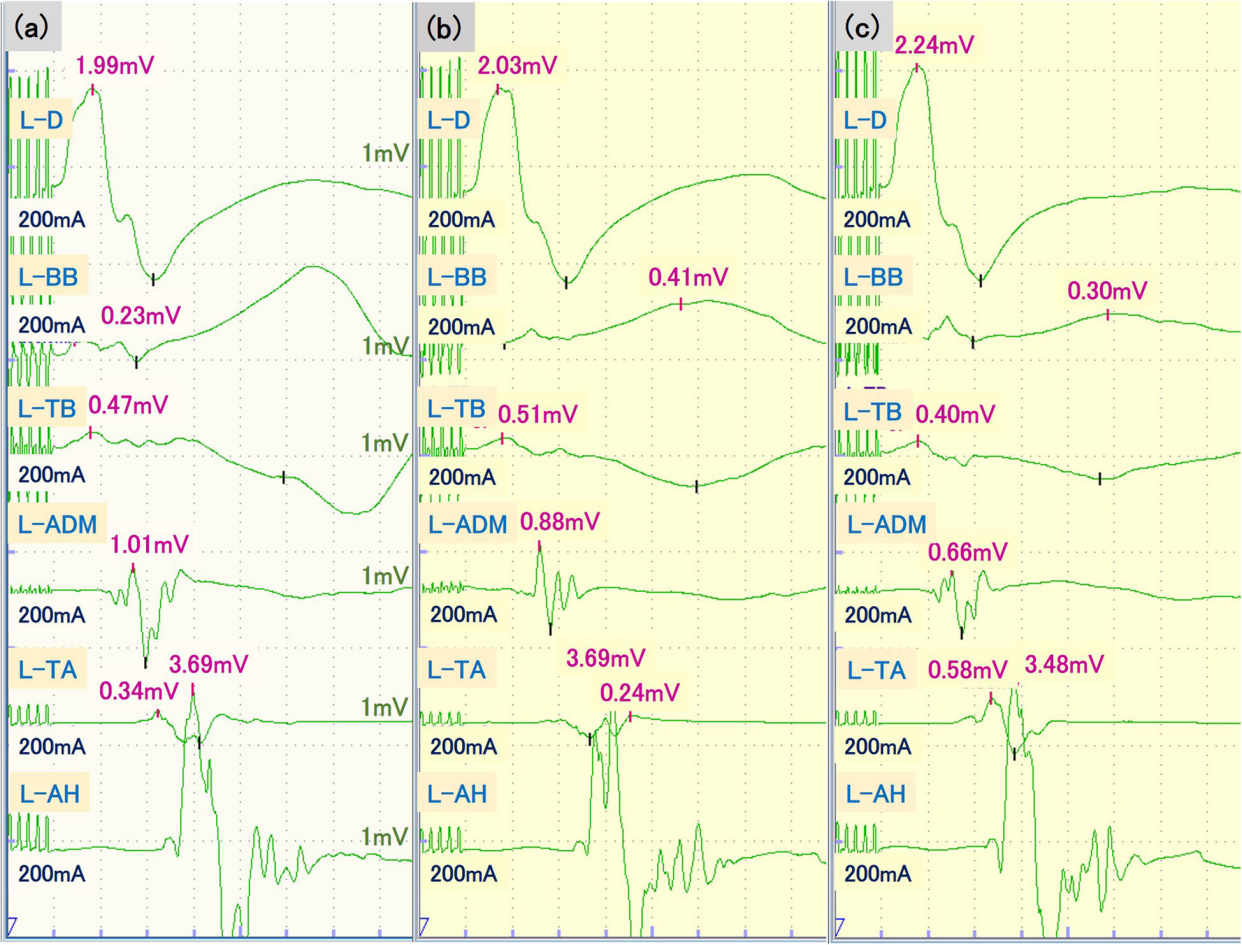
MEP responses from the left upper and lower extremities before surgery (**a**), during laminoplasty (**b**), and at the end of surgery (**c**) in case 1. Anesthesia was maintained with remimazolam 0.5 mg/kg/h and remifentanil 0.2 μg/kg/min. D, deltoid; BB, biceps brachii; TB, triceps brachii; ADM, abductor digiti minimi, TA, tibialis anterior; AH, abductor hallucis muscles

A 76-year-old woman without preoperative motor palsy was scheduled to undergo laminoplasty for cervical spondylotic myelopathy. Her medical history was significant only for obesity. General anesthesia was induced with remimazolam at 6 mg/kg/min and remifentanil at 0.3 μg/kg/min. Rocuronium (50 mg) was administered before tracheal intubation. After adequate mechanical ventilation was established, the patient was placed in the prone position. Baseline transcranial electric motor evoked potentials (MEPs) were evaluated using the Neuromaster neurophysiologic monitoring system (Nihon Kohden, Tokyo, Japan) after administration of sugammadex to antagonize neuromuscular block produced by rocuronium (Fig. 1a). Transcranial electrical stimulation by train-of-five pulses with a 2 ms interval at 500 V and 200 mA was delivered by placing two corkscrew electrodes at C3 and C4 position (international 10–20 system for electrode placement). Myogenic MEPs were recorded using subdermal needle electrodes at the upper extremities including the deltoid, biceps brachii, triceps brachii, and abductor digiti minimi muscles, as well as from the lower extremities including the tibialis anterior and abductor hallucis muscles. The amplitude of the MEPs was determined by identifying the peak-to-peak amplitude, whereas latency was measured from the start of the stimulation to the onset of myogenic activity. MEP recordings were considered successful when the recorded amplitudes were greater than 50 μV. A 50% reduction of MEP amplitude or 10% prolongation of latency was considered as significant change.

Anesthesia was maintained with remimazolam at 0.5 mg/kg/min and remifentanil at 0.2 μg/kg/min to maintain the value of entropy monitoring, an indicator of the depth of anesthesia, in the range of 40 to 60. No muscle relaxant was added after induction of anesthesia. MEP responses were recorded during the laminoplasty procedure using a surgical microscope (Fig. 1b). No significant MEP changes were observed relative to baseline values during laminoplasty. Before the end of surgery, MEP responses were recorded for final confirmation (Fig. 1c). No remarkable changes in MEP signals were observed. The operation was finished with no airway problems, and the postoperative course was uneventful.

### Case 2

**Fig. 2:**
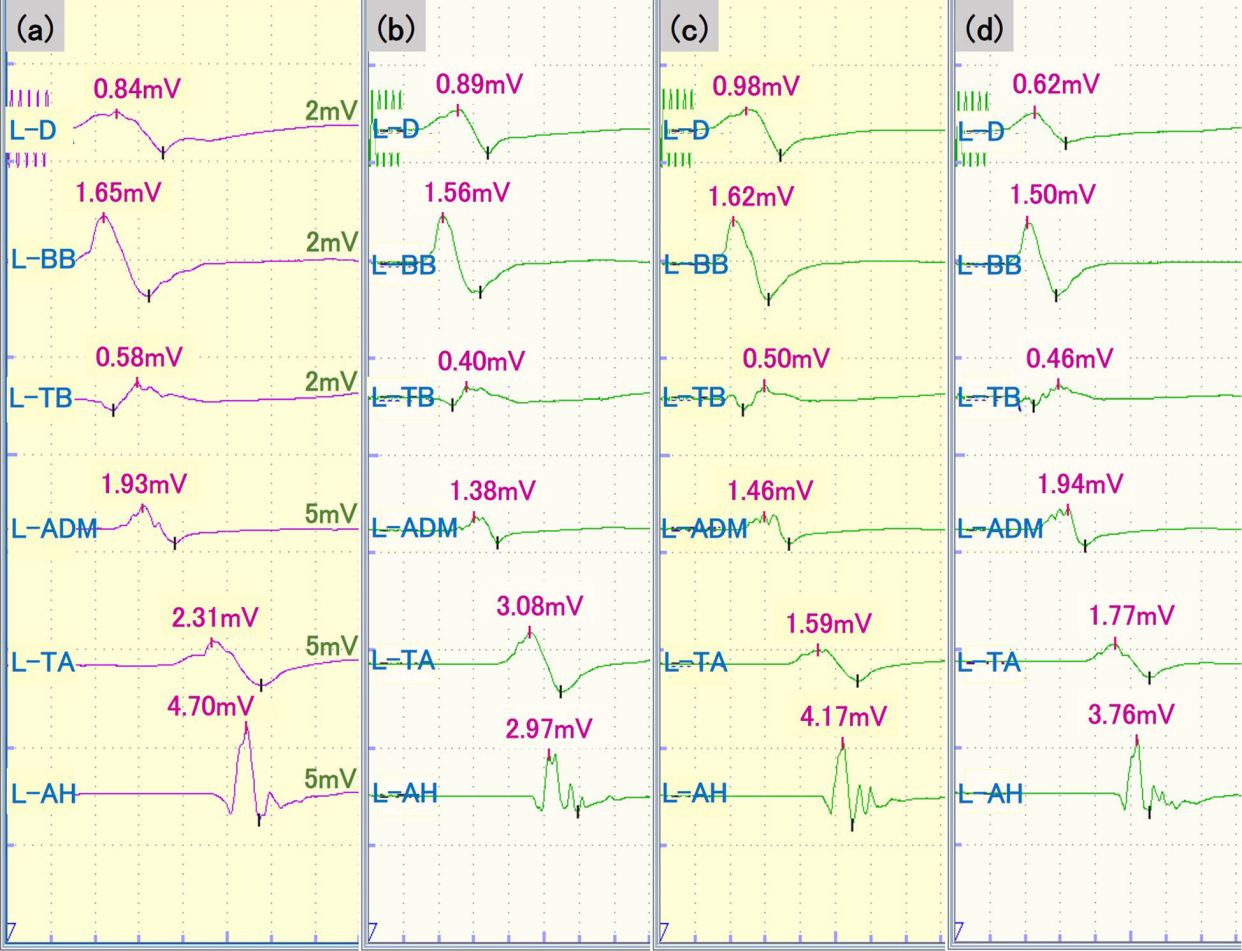
MEP responses from the left upper extremities before surgery (**a**), during laminoplasty (**b** and **c**), and at the end of surgery (**d**) in case 2. Remimazolam 0.5 mg/kg/h (**a**), 1 mg/kg/h (**b** and **d**), or 1.5 mg/kg/h (**c**) was administered with remifentanil 0.3 − 0.5 μg/kg/min. D, deltoid; BB, biceps brachii; TB, triceps brachii; ADM, abductor digiti minimi, TA, tibialis anterior; AH, abductor hallucis muscles

A 70-year-old man without preoperative motor palsy was scheduled to undergo anterior cervical discectomy and fusion for cervical myelopathy due to cervical disc herniation. His medical history was significant only for back pain. General anesthesia was induced with remimazolam at 12 mg/kg/min and remifentanil at 0.3 μg/kg/min. Rocuronium (30 mg) was administered before tracheal intubation. After the patient was placed in the prone position, baseline MEPs were recorded after administration of sugammadex. MEP monitoring protocols were similar to those in case 1. During maintenance of general anesthesia, remifentanil was used at 0.3–0.5 mg/kg/min. Remimazolam was gradually increased from 0.5 to 1.5 mg/kg/h to maintain the value of entropy monitoring in the range of 40 to 60. No muscle relaxant was added after induction of anesthesia. MEP responses were recorded throughout the operation, but no significant MEP changes were observed relative to baseline values (Fig. 2). The operation was uneventfully completed, and the postoperative course was uncomplicated.

## Discussion

Intraoperative MEP monitoring during spine surgery is used to detect neurologic deficits that occur due to surgical maneuvers, vascular injury, or spinal cord ischemia. General anesthetics, especially volatile anesthetics, induce suppression of MEP amplitude, mainly by affecting synaptic transmission [5]. Volatile anesthetics appear to suppress both synaptic activity in the brain and spinal motor neuron excitability [6, 7]. Intravenous anesthetics, including propofol and benzodiazepine, produce inhibitory effect of interneuron activity through GABA(A) receptor with minimal suppression of spinal motor neuron excitability [8, 9]. Therefore, intravenous anesthetics are preferred over volatile anesthetics for the purpose of MEP monitoring. Remimazolam is a novel short-acting benzodiazepine that has higher affinity for the GABA(A) receptor and is metabolized into a lower-affinity carboxylic acid metabolite than the conventional benzodiazepine midazolam [10]. Midazolam can produce marked depression of MEP responses [11], whereas the impact of remimazolam on MEP responses is not well-known.

In the cases described here, no significant MEP changes were observed throughout operations performed under general anesthesia using remimazolam and remifentanil. Long duration of operation or prolonged exposure to anesthetics can cause MEP responses to deteriorate, a phenomenon called “anesthetic fade” [12]. In case 1, there were no significant changes in MEP signals under general anesthesia using a fixed dose of remimazolam at 0.5 mg/kg/h and remifentanil at 0.2 μg/kg/min. Although the optimal dose of remimazolam during the maintenance phase of general anesthesia is 1 mg/kg/h [13], maintenance dose of remimazolam in this case was lower than previously reported. Remifentanil appears to synergize with the anesthetic effect of remimazolam [13]; thus, using remifentanil in combination with remimazolam enables a reduction in the required amount of remimazolam without interfering with MEP monitoring. In case 2, increasing the dose of remimazolam from 0.5 to 1.5 mg/kg/h during the operation did not affect MEP signals. Intravenous anesthetics can affect MEP responses in a dose-dependent fashion [14]. During continuous infusion of midazolam, progressive suppression of MEP signals has been observed with increasing doses [15]; in this case, however, increasing the dose of remimazolam did not affect MEP signals. Although we could not clarify the mechanism of the differences between midazolam and remimazolam on MEP monitoring, the difference in dosage may cause different effect on MEPs. Electroencephalogram (EEG) monitoring can obtain good arousal and avoid overuse of anesthetics by assessing the depth of anesthesia [16]. In these cases, it is possible that the MEP responses did not change during the operation because the amount of remimazolam was minimized by using the EEG value as an index of remimazolam dose.

This report has some limitations. First, it is unclear whether remimazolam suppresses MEP because there are no control data without it. Second, this is only an observational finding with two cases; thus, further studies are needed to consider about the indication of remimazolam for operations using MEP monitoring.

In summary, here we have reported our experience with two cases of intraoperative MEP responses during spine surgery under anesthesia with remimazolam and remifentanil. In both cases, MEP monitoring was successfully performed by either fixed or increasing doses of remimazolam. Thus, anesthesia using remimazolam and remifentanil can be a valuable alternative for spine surgery with MEP monitoring using an EEG monitor to assess the optimal dose.

## Data Availability

The data used in this case report are available from the corresponding author on reasonable request.
